# “Posting policies don’t change because there is peace or war”: the staff deployment challenges for two large health employers during and after conflict in Northern Uganda

**DOI:** 10.1186/s12960-019-0361-9

**Published:** 2019-04-17

**Authors:** Richard Mangwi Ayiasi, Elizeus Rutebemberwa, Tim Martineau

**Affiliations:** 1 ReBUILD Consortium and Department of Health Policy Planning and Management, Makerere University, School of Public Health, P.O. BOX 7072 Kampala, Uganda; 2 ReBUILD Consortium and Department of International Public Health, Liverpool School of Tropical Medicine, Liverpool, L3 5QA United Kingdom

## Abstract

**Background:**

Between 1986 and 2006, the Acholi region in Uganda experienced armed conflict which disrupted the health system including human resources. Deployment of health workers during and after conflict raises many challenges for managers due to issues of security and staff shortage. We explored how deployment policies and practices were adapted during the conflict and post-conflict periods with the aim of drawing lessons for future responses to similar conflicts.

**Methods:**

A cross-sectional study with qualitative techniques for data collection to investigate deployment policy and practice during the conflict and post-conflict period (1986–2013) was used. The study was conducted in Amuru, Gulu and Kitgum districts in Northern Uganda in 2013. Two large health employers from Acholi were selected: the district local government and Lacor hospital, a private provider. Twenty-three key informants’ interviews were conducted at the national and district level, and in-depth interviews with 10 district managers and 25 health workers. This study focused on recruitment, promotions, transfers and bonding to explore deployment policies and practices.

**Results:**

There was no evidence of change in deployment policy due to conflict, but decentralisation from 1997 had a major effect for the local government employer. Lacor hospital had no formal deployment policy until 2001. Health managers in government and those working for Lacor hospital both implemented deployment policies pragmatically, especially because of the danger to staff in remote facilities. Lacor hospital introduced bonding agreements to recruit and staff their facilities. While managers in both organisations implemented the deployment policies as best as they could, some deployment-related decisions could lead to longer-term problems.

**Conclusion:**

It may not be possible or even appropriate to change deployment policy during or after conflict. However, given sufficient autonomy, local managers can adapt deployment policies appropriately to need, but they should also be supported with the necessary human resource management skills to enable them make appropriate decisions for deployment.

## Background

There has been a major focus on meeting the health workforce needs to achieve Universal Health Coverage (UHC) [1, 2]. Distribution of the health workforce is key to ensure coverage is “universal”. The relevant human resource management function for ensuring equitable distribution is “deployment”. Much work has been done on developing incentives to support deployment [3, 4], but the focus here is on a bundle of human resource management (HRM) practices [5] to support equitable deployment of health workers including recruitment and promotion, transfers and bonding (see Table 1).

**Table 1:** Human resource management functions and events related to deployment

**Fig. 1:**
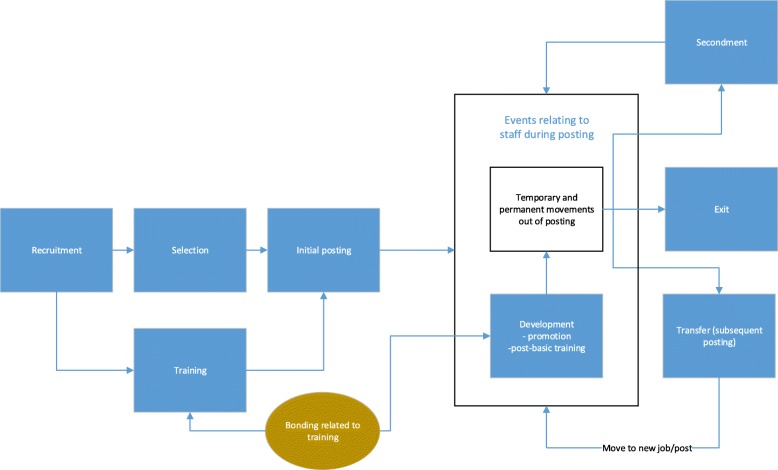
Human resource management functions related to deployment

The term “deployment” is frequently used in relation to the distribution of staff, but rarely defined. One of the few definitions can be found in the Canadian Public Service Employment Act which states that deployment means “the transfer of a person from one position to another” [6]. An alternative definition given by the HRH Global Resource Centre is “how health workers are assigned to different posts or how the distribution of personnel is determined”.^a^ We interpret deployment as including all the relevant human resource management functions: recruitment, selection (which for those entering public service and being funded by the government often takes place on entry into pre-service training), initial posting (often linked to bonding). Once the staff member is in post, there may be development activities including post-basic training and promotion. Both will have implications for staff movements: post-basic training may lead to promotion or the individual seeking a higher-level role; promotion usually involves a change of post and possibly a change of location if higher-level positions are not available locally. Other possible movements from the initial posting include: secondment, or deputation, to another post (normally temporary with the person returning to the original posting); transfer to another position (either decided by management or granted at the request of the individual); or exit from the organisation (resignation to take up another job, retirement or dismissal). These functions and movements are represented in Fig. 1.	

^a^ http://www.hrhresourcecenter.org/node/2985

Despite the importance of deployment policy (or “formal rules for decision-making authority”) and practices for large health service employers, relevant literature is limited—especially in low- and middle-income countries (LMIC). Recent literature on posting and transfer covers similar ground [6–11] and highlights discrepancies between policy and practice. However, this literature is based on stable contexts, including India and Ghana, as referenced above. Deployment may face greater challenges, but have a greater role for achieving UHC in conflict and post-conflict situations where staff are scarce and concentrated in urban areas [12].

In Acholi sub-region in Northern Uganda, a protracted armed conflict took place between 1986 and 2006. Many people moved to internally displaced people’s (IDP) camps scattered across the districts of Amuru, Kitgum, Pader, Agago and Gulu—the main area of conflict [13]. Health centres were closed because of insecurity and some of them relocated to the IDP camps, run by non-governmental organisations (NGOs), while supervision by district health teams was limited. Health professionals were equally displaced into larger trading centres; some fled the rural areas where the effects of the war were more direct, while others migrated to more stable, neighbouring districts. Lacor hospital, a faith-based health provider in Gulu district, constructed barrier walls around the main hospital and the satellite health centres in order to provide security and to retain health workers at the facilities. When the insecurity intensified, satellite centres of Lacor were also temporarily closed and staff were redeployed to the hospital until the security situation normalised.

Since mid-2006, peace has returned to the region. Resettlement of displaced populations has taken place [14], the reconstruction of health centres began and the recruitment and deployment of new health professionals started. The period of conflict and post-conflict provides the opportunity for understanding how managers applied available human resource policies under a constrained situation [14].

This paper examines how two different health services providers managing multiple facilities (district-level government health offices and one faith-based hospital with three satellite clinics all of level III) adapted their policies and practice on deployment to the changing context of the conflict and post-conflict period in Northern Uganda. The lessons derived should be useful in helping health service providers be more adaptable to changing contexts in Uganda, and other conflict-affected states, and complement findings from a similar study on health worker incentives conducted within the same research programme [15].

## Methods

The aim of this study was to identify changes in deployment policies and practice during the conflict (1986–2006) and post-conflict period (2006–2013). Two employers with multiple facilities that required staff to be transferred between them were selected to identify similarities and differences in approaches and challenges. The employers selected were the district-level government health providers and Lacor hospital—a private-not-for-profit (PNFP) institution. District local government and one other local employer were purposively selected because they were large employers in the health sector capable of moving health workers around within the system. This was a cross-sectional study that used a retrospective approach, reviewed documentation on the deployment policies and obtained qualitative data from study respondents. This study was conducted in the post-conflict region of northern Uganda. The study sites Amuru, Gulu and Kitgum districts were purposively selected because they represent the epicentre of the conflict in Northern Uganda.

### Study setting

Gulu and Kitgum districts existed before the start of the conflict, while Amuru district was carved out of Gulu district in 2006. All three districts are located within the Acholi sub-region in Northern Uganda. Distribution of health centres and district population is summarised in Table 2. Lacor hospital was founded in 1959 and is considered to be the largest PNFP hospital in Uganda with a bed-capacity of 482 [16]. Lacor hospital is based in Gulu district where it operates two satellite health centres in Opit and Lalogi and a third in Pabo in Nwoya district to the west of Gulu district [17]. In this paper, “Lacor hospital” refers to both the hospital and its satellites.

**Table 2:** Number of health facilities, their levels per district and total district population [53–55]

Level of care/district	Kitgum	Gulu	Amuru	Lacor
II	14	48	23	00
III	08	14	07	03
IV	01	02	01	00
Hospital	02	04	00	01
Total facilities	25	76	31	04
Total population	256 000	407 500	242 300	n/a

Level II provides mainly out-patient services. Level III provides out-patient and in-patient services. Level IV provides out-patient, in-patient and emergency obstetric care services

### Study populations

The study population included policy-makers, health service managers and health workers in order to get perspectives both on policy and practice. Study respondents were selected from the public sector and Lacor hospital. For the public sector, respondents were drawn from the national, district and health facility levels, while for Lacor hospital, respondents were drawn from the hospital as well as its three satellite sites. National- and district-level respondents were selected on the basis of their position and its bearing on human resource-related issues. Health workers were selected to include staff that had practised through the conflict and the post-conflict period.

### Data collection

Data collection was conducted in the following sequence: first, document review, followed by central-level interviews, then district-level interviews. District-level interviews including Lacor hospital were conducted in parallel. Document review was conducted by searching for policy documents containing relevant sections related to deployment (appointment, post basic training, bonding and transfers). The documents reviewed were purposively selected and they included the Public Service Standing Orders [18], Employment Manual for St Mary’s Hospital [19], Scheme of Service [20] and the Human Resource for Health Policy in Uganda [21]. These documents were selected because they provide policy information on deployment which is the focus of this study. Data were obtained by key informant interviews (KII) with policy-makers to help clarify the documented information on the deployment policies and practice and to identify contextual factors that may have influenced development of the policies or implementation of the systems. In-depth interviews (IDI) with managers explored their perceptions and experiences of implementing the deployment policies and systems during the study period, while the IDIs with health workers, which included career histories with timelines, explored their perceptions and experiences of the deployment policies and practices and their evolution over time. Interviews were conducted between June and December 2013. Health workers at the health facility were interviewed about their entire career from its start to the point of data collection [22]. The information gathered spanned from 1986 to 2013 dichotomised as conflict (up to 2006) and post-conflict period. A total of 58 respondents were interviewed including policy-makers, managers and health workers (see Table 3).

**Table 3:** Respondent details

Data collection method	Central (female)	Kitgum (female)	Gulu (female)	Amuru (female)	Lacor (female)
KII policy-makers	9 (3)	5 (2)	4 (0)	5 (1)	0
IDI managers	0	3 (0)	2 (0)	2 (0)	3 (1)
IDI health worker by cadre					
Doctor	0	1 (0)	1 (0)	1 (0)	0 (0)
Clinical officer	0	2 (0)	2 (0)	2 (1)	1 (0)
Nurse	0	1 (0)	2 (1)	3 (2)	2 (2)
Midwife	0	2 (2)	2 (2)	1 (1)	2 (2)
Total	9 (3)	14 (4)	13 (3)	14 (5)	8 (5)

### Data analysis

Analysis of data from document review was based on content analysis [23]. The main output was a narration of the evolution and description of the deployment policies and practices based on pre-determined sub-themes. The sub-themes included recruitment, initial posting, post basic training, bonding and transfers. These sub-themes were used to summarise the data throughout the conflict and post-conflict period. The sub-themes related to deployment were later categorised into three thematic areas [24, 25]: “entry into the system” (recruitment, selection and initial posting); “development” (post-basic training and promotion); “subsequent movements” (transfers); bonding which was integrated into the sections on both initial and post-basic training. For key informant interviews, in-depth interviews and career histories, participants’ responses were coded and grouped by themes related to the research questions. Thematic analysis was used with the assistance of Atlas.t^1^ software. Summaries for the sub-themes produced in the thematic analysis were collated into a spreadsheet first by district (district KIIs and in-depth interviews). A summary by topic was then created across the three districts and with the addition of the national KII summaries and data from the document review. A single step was required for summarising different data sources for Lacor hospital. Finally, summaries for the local government (LG) employers and Lacor hospital were separately compiled for comparison. Results were described by synthesising how the different themes were practised during and after the conflict period and between local government and Lacor hospital.

### Ethical considerations

This study was approved by the Liverpool School of Tropical Medicine, UK, Makerere University School of Public Health, Uganda, and the ethics committee at Lacor hospital. The study was registered with Uganda National Council for Science and Technology (UNCST). Interviewed respondents first gave their written consent before participating in the study. Data access was restricted to the researchers and only used for research purposes.

## Results

We first present findings on the broader policy environment in the public health sector and Lacor hospital health provider according to the document review and KIIs. Against that background, we present the changes in policy and practice related to deployment over the study period.

Our findings show that, over the period covered by the study, broad changes took place in the policy environment both government-run health services at the district level and at Lacor.

Prior to the introduction of decentralisation from 1997, all public service employees were managed using the public service handbook from the 1960s [18]. Decentralisation was introduced nationally during the period of the conflict in the North. The Local Government Act provided a framework for the devolution of powers including the autonomy for management of human resources (HR) to local governments [26]. The Local Government Act empowered district service commissions (DSC) to recruit (advertise, shortlist and interview potential candidates). Other HRM functions (funding of posts, deployment) were managed by the Chief Administration Office (CAO) for service departments such as the Department of Health. The Public Service Standing Orders [18] are still used by the HR department at the district level, though the implementation—such as recruitment and transfer—has moved to the district level.

In Lacor hospital, before the conflict period and to 2001, most HRM procedures were conducted informally. Subsequently, administrative reforms were implemented. In order to be able to access government grants, faith-based organisations (FBO) were required to use formalised recruitment procedures. The Uganda Catholic Medical Bureau (UCMB) produced a template for a human resource management manual which was adopted by Lacor hospital, initially in 2001 and revised in 2010 [19, 27]. A fully-fledged HR unit headed by a Human Resource Officer was also established at Lacor hospital.

In the following sections, we turn to describing in more detail the policy and practice of deployment as described in Table 1, and changes between conflict and post-conflict periods. The key question about the change in policy was answered by a central-level key informant: “Posting policies don’t change because there is peace or war. They remain the same.” This finding is supported by other data collected, but the key changes were with the way in which policies were implemented. These findings are reported, for each of the two employers, under the following headings: recruitment and selection, initial posting, transfers, post-basic training and promotion; bonding is integrated into the sections on both initial and post-basic training. Unusually, there was no evidence of the use of secondment, as shown in Fig. 1, by either employer.

### Recruitment and selection

#### Local government policy and practice

Prior to decentralisation, the policy was that centralised recruitment was conducted by the Public Service Commission (PSC). Interviews were carried out centrally by the PSC. Following decentralisation, the policy was for local recruitment by the District Service Commission. There was no policy on bonding after recruitment.

Though the responsibility for recruiting staff had been shifted to local government, during the conflict, this was difficult due to the challenge of advertising posts and for reason of security.

A review of the immediate post-conflict situation in the Acholi region in 2007 conducted as part of the Peace Recovery and Development Programme (PRDP) identified severe shortages of health workers to population (1:4000 compared to 1:2000 in West Nile) and recommended accelerated recruitment with central support with the addition of incentives [28]. The challenge was to rebuild the workforce in the region taking advantage of the opportunities provided by decentralisation.

… right now, with improvement of security, those policies are now being implemented [referring to recruitment policy], … when you come to health workers, there is that liberty now, we recruit health workers locally (Local government official, Amuru district).

Though some NGOs exacerbated the shortages of government staff by recruiting them for their own programmes, other NGOs initially supported the recruitment process by financing advertisements. Some NGOs and projects even temporarily funded LG staff.

We have a few NGOs helping us to pay up the health workers’ wage - they recruit using the District Service Commission and then they pay up. This is boosting our human resource. (Local government official, Amuru district)

Selection was carried out using the public service regulations. However, under decentralisation, these practices were considered less transparent, where officers in charge could hire inadequately qualified relatives and friends. This could have compromised quality of health care provided.

they have abused the meaning of decentralisation. [ ….] it’s very difficult for an individual not from the district to benefit from the recruitments because of nepotism and sectarianism they cannot attract the right people to work. (Personnel officer, Amuru)

It was not until 2012 that central government intervened through the introduction of temporary policy of accelerated recruitment that was carried out with donor support. However, this was nationwide rather than specifically for the Acholi region [29]. Though supportive, the centralised recruitment process did not sit well with decentralised structures. For example, some staff made applications to more than one LG. Due to lack of coordination, candidates would get multiple appointment letters from different districts, many of which were unable to fill their vacancies.

I submitted job applications to Amuru and Kitgum district. I wasn’t sure of getting a job, so I tried both districts but good enough I succeeded in both districts. Yes. They called me for both and I did the interviews. (Nurse, Kitgum district)

#### Lacor policy and practice

During the conflict and prior to 2001, there was no clear written policy on recruitment and selection in Lacor. The handbook of 2010 states that:

...selection of a candidate is based both on the academic qualifications and on the level of experience …. consideration … to written references of past and present employers, previous professional experience, … the candidate’s behaviour and conduct at the time of the interview ([19]: page 6)

In practice, recruitment of nurses was carried out at the enrolment stage (see Fig. 1) for courses run by Lacor hospital. Local residents were targeted, since they were more likely to remain in service. During the conflict, when staff were in especially short supply—partially because of the arrival of other employers—students were sponsored for training with funding from DANIDA (Danish International Development Agency) and therefore bonded to service after qualification. Bonding was considered important during the conflict because of the low numbers of staff and also low enrolment numbers in training school.

In Kalongo, once the NGO [AVSI] pays for you, after your study, you first work in Kalongo for two years. That is more of compensating for what the NGO has done for you, after that you are free to look for other jobs (Midwife Kitgum district)

Lacor used additional strategies to address staffing shortages. They were able to recruit additional staff through the Catholic Church within the diocese. If the medical superintendent identified a nun trained in nursing/midwifery, he could then request the diocesan Superior General to deploy the nun to the hospital. Another strategy, a form of task-shifting, was using enrolled comprehensive nurses to address the shortage of midwives.

Following the end of conflict when enrolment numbers increased, the use of bonding to retain health workers became less prevalent. The emphasis shifted to recruiting the better students from the training courses run by Lacor hospital.

### Initial posting

#### Local government policy and practice

In the first part of the conflict period, initial posting following recruitment was managed by the Ministry of Public Service. The policy was that staff went wherever they were posted in the country.

Those who had been in post for many years indicated that the policy was implemented and staff were indeed centrally recruited and posted to any health facility in any district in the country.

For us the two of us who did the course together, we were posted to Lira Regional Referral Hospital. We did not choose to go there; it was just normal posting from the Public Service Commission. (Doctor Amuru district)

The major policy change was after decentralisation whereby the LG posted staff where they were needed within the district. However, these benefits could not be realised, as there was little recruitment during the conflict period. The decentralised practice of initial posting was only relevant in the post-conflict period.

Always when you pass the interview, it’s the head of department [in this case the District Health Officer] to post you in a vacant place where you should go and work. It’s not you to choose because you have already written the acceptance letter committing yourself to the job description of that job you have been employed to go and do. (Clinical officer, Amuru district)

This would have enabled managers to post staff according to need.

#### Lacor policy and practice

In Lacor, during conflict, staff were notified of their initial postings made through a general list displayed in the institutions where they were receiving training, bypassing any selection process (see Fig. 1).

Ok they make a list and send it to the training school yeah, then from the training school the principal will tell you that you have been taken you are going to remain and work in this particular ward also they will send the report to the in-charge then the in-charge will welcome you … (Midwife Lacor hospital)

In the middle of the conflict ([30]: page 7) and after, Lacor hospital established a fully fledged HRM unit and the recruitment, appointment and transfer process started to mimic the government system as already shown under the section “Recruitment and selection” above.

### Transfer

#### Local government policy and practice

Both the past and more recent standing orders lay out the rules on transfers, but no adjustments were made to address problems created by the conflict. In the early conflict period, transfers were made across the country. Since decentralisation, transfer relates to movements within a district, for the rules are “such deployment will be in public interest and on recommendation of the responsible officer, not a punitive or disciplinary measure”. ([18]: page 27). Staff who do “not comply with the posting instructions will be liable to disciplinary action.” ([18]: page 99). Regarding the length of the posting, it states that “a public officer may be transferred after a continuous stay in his/her station for at least three years and not exceeding five years”.

In practice during the conflict period, managers realised it would be difficult to expect staff to move to areas of insecurity. Most health centres were abandoned. The few that survived were staffed by a small number of health workers and therefore it was difficult to move staff around and managers did not want to risk losing any by making unpopular transfers,

Ok, well during the insurgency period certainly there were a lot of system break downs and families were disintegrated, you find that during that period, people were in confined places in the IDPs [internally displaced peoples camps]. So, it was a bit difficult for health workers to even accept to be deployed in areas which are affected by war so because you don’t know what’s happening, people fear for their lives. (Manager, national level)

In Gulu, transfers were minimal and staff were encouraged to work in the same duty station. When posting staff, the District Health Officer applied the guidelines for transfers but also used his ingenuity to post good performers together with those who were performing less well. District managers said they tried to be sensitive when posting. For example, in Gulu, couples were sometimes deliberately not posted together during the conflict. This was done to preserve the family unit in case of a rebel attack, as it was hoped that at least one partner would therefore survive the war and be there to take care of the children and family.

In the post-conflict period, the transfer process was normalised. In Gulu, couples were once again allowed to work together. In Amuru and Kitgum, special considerations were made for illness amongst staff members and they were posted nearer to the hospital for quicker medical attention. However, managers were not rigorously following the rules regarding the length of posting. It was reported that some staff were left in the same posting for up to 5 years and this was said to be affecting retention. This might be because staff felt trapped in their postings [31].

#### Lacor policy and practice

Being a small organisation, Lacor did not have a formal policy for transferring between its units during the conflict. However, with the development of their overall HRM policy in 2010, transfer was included: “… the Hospital reserves the right to deploy the employee to its peripheral units on prolonged or permanent basis.” ([19]: page 8). A period of 2 years was stipulated for the postings.

In practice, during the conflict, Lacor hospital managers negotiated with health workers willing to continue serving at the satellite health centres. The hospital also provided a hardship allowance for staff that were serving in satellite health centres to cover transport costs to enable them to come to town or to hospital for other business such as collection of salaries.

After the conflict, some staff negotiated with managers to remain in the satellite health centres for longer than the stipulated 2 years and this request was normally accepted. Married staff with children were given special consideration when affecting transfers. As the staff members said, when they were with their families, transfers were unlikely.

yeah there are very many people who have refused to come to health centres [because of] issues of family. (Clinical officer, Lacor hospital)

### Post-basic training and promotions

#### Local government policy and practice

As is usually the case in large organisations, a higher qualification is needed for promotion and the availability of a vacant post at a higher level: “attainment of higher qualifications does not automatically qualify a public officer for promotion to the next grade … such an officer who has attained a higher qualification is eligible to a higher grade when a vacancy exists but the said officer must compete with other eligible candidates” ([18]: page 154).

There were variations in the implementation of promotion policy by managers across the districts. In Gulu, it was reported that it was possible to continue the process of promotion during the conflict period. In Kitgum, managers waited until the end of the conflict before first seconding staff for further training and then, in recognition of their resilience to service during the conflict, they were subsequently given special consideration for promotions. Though this was intended as a reward, these HRM decisions could be counterproductive to the district managers. Some staff did not return after their training both because the bonding policy was not effective and staff become over-qualified for their current posts or were now much stronger candidates for new jobs. In Amuru, post-conflict training was clearly seen as an exit route as the district had no senior posts to offer for promotion:

Training is an exit route. But of course, when somebody starts to specialise, then he becomes over-qualified to work in a district. He needs to go to a referral hospital and that has been a big challenge to us. (Manager, Amuru district)

#### Lacor policy and practice

The training policy of 2010 states the following: “In case the employee intends to join a training programme, it shall be at the discretion of the Hospital Executive Director to grant an extended leave without pay, if he recognises that the Hospital might in future benefit from the additional qualifications acquired by the employee. In this case the employee shall retain his/her job and can come back to it at the end of the unpaid leave.” ([19]: page 22).

In practice, this policy was implemented flexibly, and staff appear to have been given “leave-with-half-pay” and reinstated on full pay on completion of training.

## Discussion

In this paper, we have attempted to show how two different employers with multiple health facilities adapted their policies and practice on deployment to the changing context of the conflict and post-conflict period in Northern Uganda spanning the period between 1986 and 2013. Though the nature and size of the two organisations were different, it was possible to see how both adapted practice during the conflict and in the immediate post-conflict period. We have focussed on policies that were documented, though unwritten policies [32] may have been in use. We planned to use routine staffing data to identify changes in health worker availability, distribution and attrition during the study period. However, as other researchers have found in these contexts [33–35], it is difficult to find sufficiently complete data making relevant comparisons impossible to analyse. Instead, we relied on career histories with timelines (as we have in other research in these contexts [22]) and interviews with managers. Respondent recall over such a long period was an inevitable challenge but, where possible, triangulated our data sources [33, 34] Nevertheless, we may not have identified all aspects to the change in deployment policy and practice.

As context is a key factor in policy-making [36], it is reasonable to question whether the conflict in the Acholi might lead to a change in policy. This was clearly not the case regarding deployment policy for government-run health services at the district level and the major changes in deployment policy were created by the overarching policy of decentralisation. In Lacor hospital, there were no formal deployment policies during the conflict and more formal HR policies were brought in as part of wider reforms.

Nevertheless, the study identified more interesting changes in deployment at the implementation level. Whereas the literature shows frequent discrepancies between policy and practice in relation to deployment [7, 9, 11, 37, 38], in the absence of clear policy response to the conflict, managers appear to have made the best use of their available decision space [39, 40] to address the challenges of deployment.

During the conflict period, managers at Lacor hospital were able to be more resourceful in the recruitment process focussing on recruitment into nursing training provided by their institution and thereby having a ready supply of new staff and linking this to a bonding system. While the implementation of bonding policies is often problematic [41], this can be effective in smaller organisations. The potential benefits of recruitment in a decentralised system [42] could not be realised by LG during conflict. While NGOs are often competitors for government in these contexts [43], LG was able to get timely support from NGOs and development partners (DPs) for funding the recruitment process and salaries. This was done on a national scale in post-conflict Liberia after some delay [44]. Even after the end of the conflict, LGs faced the common challenge in decentralised contexts of getting enough qualified applicants as reported in Tanzania [45]. There was also a delay of some 6 years for the central level support in Uganda, and this was a national initiative not specifically for the Acholi region in spite of the Peace Recovery and Development Programme’s (PRDP) recommendations about the need for “accelerated recruitment” in 2007.

In the context of security for health workers and the risk of discontented health workers leaving, employers were sensitive in their handling of transfers demonstrating some basic appreciation of labour market factors [46] and seem to have shown both compassion using “emotional intelligence” [47].

The examples of collaborating with partners to employ staff and addressing staff needs to improve retention demonstrate the managers in both organisations were effectively using their available “decision space”—in some cases informal [48] — to adjust to the changing context. This was easier for Lacor hospital being a smaller institution which has integrated functions for recruitment, deployment and funding of posts, has the autonomy to make rapid changes and has strong links to training institutions to support their recruitment processes.

In contrast, at the local government level, the HR functions are split between the DSC and the CAO. Moreover, government training institutions are under the Ministry of Education and therefore can act less nimbly than Lacor hospital. While the problem in human resource management is often the lack of “implementation fidelity” [49] between policy and practice, in the case of these two employers, the discrepancy appeared to have positive outcomes. Perhaps the emphasis should therefore be less on adapting policy to changing contexts, in particular for organisations governed by national regulations and the change in local context. The focus should be more on ensuring local managers have the skills to adapt to changes in their local contexts as highlighted in the immediate post-conflict period in South Sudan [33]. The study identified examples of positive adaptation of policies, but there were examples of potential unintended negative longer-term consequences of decisions made, particularly in LGs: for example, sending for training as a reward leading to loss of staff, or leaving staff in a post too long. This can be avoided with the skills of developing a balanced bundle of HRH practices [50]. It would not be surprising if managers at the district level did not have the relevant skills, as training in HRM is rare even for senior managers in Africa [51].

A strong health workforce is critical to building resilient health systems [52]. The following lessons may be useful in ensuring a strong health workforce both during and after conflict or crisis.

1. Smaller organisations are likely to be able to nimbly adapt both policy and practice to changing contexts. Larger organisations may find this more difficult, especially if the challenges are more localised. In this situation, emphasis should be on supporting local managers to adapt the implementation of deployment—and other HR practices—within or even beyond their normal decision space.
2. While support as the context changes would be desirable, preparation as part of general management training would be more effective and allow for quick reactions. An essential part of that training would be the skills of developing a balanced bundle of HRH practices. Representations of the complexity of HRH practices as provided in Fig. 1 may be useful.

## Conclusions

In the face of conflict and the early recovery period, the government did not adapt its deployment policies. In Lacor hospital, policy only became formalised sometime after the end of conflict. However, in both organisations, local managers adapted the implementation of deployment policies in the best way they could. Perhaps in future as the focus for preparing for different kinds of crises, local managers should be provided with better skills in HRM to guide their responses and support from local NGOs and development partners.

## Abbreviations

FBO: Faith-based organisation
HR: Human resources
HRH: Human resources for health
HRM: Human resource management
IDI: In-depth interviews
IDP: Internally displaced people
KII: Key informant interviews
LG: Local government
LMIC: Low- and middle-income countries
NGO: Non-governmental organisation
PNFP: Private-not-for-profit
PRDP: Peace Recovery and Development Programme
PSC: Public Service Commission
UCMB: Uganda Catholic Medical Bureau
UHC: Universal Health Coverage
UK: United Kingdom
UNCST: Uganda National Council for Science and Technology

## Footnotes

1
www.atlas.ti.com
